# Concordance between self-reported sleep and actigraphy-assessed sleep in adult survivors of childhood cancer: the impact of psychological and neurocognitive late effects

**DOI:** 10.1007/s00520-021-06498-x

**Published:** 2021-08-26

**Authors:** Margaret M. Lubas, Mariana Szklo-Coxe, Belinda N. Mandrell, Carrie R. Howell, Kirsten K. Ness, Deo Kumar Srivastava, Melissa M. Hudson, Leslie L. Robison, Kevin R. Krull, Tara M. Brinkman

**Affiliations:** 1 Department of Epidemiology and Cancer Control, St. Jude Children’s Research Hospital; 2 School of Community and Environmental Health, Old Dominion University; 3 Department of Pediatric Medicine, St. Jude Children’s Research Hospital; 4 Department of Medicine, Division of Preventive Medicine, University of Alabama at Birmingham; 5 Department of Biostatistics, St. Jude Children’s Research Hospital; 6 Department of Oncology, St. Jude Children’s Research Hospital; 7 Department of Psychology, St. Jude Children’s Research Hospital

**Keywords:** actigraphy, childhood cancer survivors, late effects, measurement concordance, self-reported sleep

## Abstract

**Purpose:**

To examine self-reported (30-day) sleep versus nightly actigraphy-assessed sleep concordance in long-term survivors of childhood cancer.

**Methods:**

477 participants enrolled in the St. Jude Lifetime Cohort [53.5% female, median (range) age 34.3 (19.3–61.6) years, 25.4 (10.9–49.3) years from diagnosis] completed the Pittsburgh Sleep Quality Index and ≥3 nights of actigraphy. Participants had neurocognitive impairment and/or a self-reported prolonged sleep onset latency (SOL). Self-reported 30-day sleep and nightly actigraphic sleep measures for sleep duration, SOL, and sleep efficiency (SE) were converted into ordinal categories for calculation of weighted kappa coefficients. General linear models estimated associations between measurement concordance and late effects.

**Results:**

Agreements between self-reported and actigraphy measures were slight to fair for sleep duration and SOL measures (*k_w_*=0.20, *k_w_*=0.22, respectively, p<0.0001), and poor for SE measures (*k_w_*=0.00, p=0.79). In multivariable models, severe fatigue and poor sleep quality were significantly associated with greater absolute differences between self-reported and actigraphy-assessed sleep duration (*B*=26.6, p<0.001; *B*=26.8, p=0.01, respectively). Survivors with (versus without) memory impairment had a 44-minute higher absolute difference in sleep duration (*B*=44.4, p<0.001). Those with, versus without, depression and poor sleep quality had higher absolute discrepancies of SOL (*B*=24.5, p=0.01; *B*=16.4 p<0.0001, respectively). Poor sleep quality was associated with a 12% higher absolute difference in SE (*B*=12.32, p<0.0001).

**Conclusions:**

Self-reported and actigraphic sleep demonstrated discordance in our sample. Several prevalent late effects were statistically significantly associated with increased measurement discrepancy. Future studies should consider the impacts of late effects on sleep assessment in adult survivors of childhood cancer.

## Background

Sleep health is an emerging topic in the cancer survivorship literature. Sleep disturbances are highly prevalent among both survivors of adult[1] and childhood onset cancers[2] and can comprise of a range of disorders including sleep-disordered breathing[3], insomnia[4], hypersomnia/narcolepsy[5], and sleep complaints such as, poor sleep quality[6], and sleep variability[6]. Importantly, sleep disturbances have been associated with decreased quality of life[7], increased emotional distress[6, 8], increased pain[7, 8], and increased risk of mortality[9] among cancer survivors.

The measurement of sleep is complex and can include polysomnography, multiple sleep latency testing, actigraphy, sleep diaries, self-reported scales, or single-item questions. While polysomnography is considered a useful measurement tool for clinical sleep assessment, its cost and feasibility are often prohibitive in large research samples or designs with repeated measures. Self-report and actigraphy are often used to assess sleep in research and clinical settings, though little is known about the performance of these measurement approaches in cancer survivors. Correlations between subjective and objective sleep measures have been examined in several clinical[10–12] and community samples[13–15], though these outcomes have not been extensively studied in survivors of adult or pediatric onset cancers. Studies comparing actigraphy-assessed sleep to self-reported sleep in large community samples of adults have reported moderate correlations between these measures[16, 13]. However, several studies across patient and community populations acknowledge potential discrepancies between self-reported sleep and actigraphy-assessed sleep[12, 16, 17, 13]. While findings of measurement discordance have been consistent, directional differences (i.e. the degree to which self-reported sleep is greater or less than actigraphy-assessed sleep) can vary by individual characteristics. In large community samples, on average, self-reported sleep duration has been found to be longer actigraphy-assessed sleep duration[13, 14, 16, 15]. However, the directional difference and/or magnitude of difference between self-reported and actigraphy-assessed sleep have been found to vary by psychosocial factors, health, and sleep characteristics of study participants[13, 16, 15].

Greater discrepancies between self-reported and actigraphy measures of sleep have also been described in medically complex patients[18] or individuals with poor sleep quality[19] relative to healthy populations. Childhood cancer survivors are at risk for several medical late effects due to cancer diagnosis and treatments[20], including psychological distress[21], sleep disturbances[6, 2] and neurocognitive impairments[22, 23], each of which may potentially impact a survivor’s experience or recall of his/her sleep. Thus, research is needed to examine the concordance between actigraphy-assessed sleep and self-reported sleep among cancer survivors who may experience a high and variable burden of chronic health conditions[20]. To our knowledge, neither the concordance between self-reported sleep and actigraphy-assessed sleep in adult survivors of childhood cancer nor the factors potentially associated with measurement agreement or disagreement in this population have been investigated. Clarifying factors associated with differences between actigraphy and self-reported sleep in survivorship may help inform study design and measurement approaches. The present study aimed to examine concordance between self-reported (PSQI-assessed) sleep for the past 30 days and nightly actigraphy, as well as associated late effects among survivors of childhood cancer. We hypothesized that actigraphy and PSQI-assessed sleep measures would be discordant and that common neurocognitive and psychological late effects of childhood cancer would be associated with greater discordance between measures. Late effects examined included fatigue, depression, anxiety, poor sleep quality, and neurocognitive impairment.

## Methods

### Participants

Self-reported and actigraphy sleep data were obtained from a sample of participants enrolled in the St. Jude Lifetime Cohort Study (SJLIFE)[24] who had been recruited to participate in a sleep and cognition intervention. SJLIFE is an ongoing retrospective cohort with prospective follow-up that was initiated in 2007 and includes childhood cancer survivors who were treated at St. Jude Children’s Research Hospital (SJCRH). Eligibility for the intervention study included survivors ≥18 years of age, ≥10 years from diagnosis, fluency in English, an IQ >79, the presence of a neurocognitive impairment and/or a prolonged sleep onset latency (see Supplemental Table 1 for a detailed list of intervention inclusion/exclusion criteria). Initially, 3348 potentially eligible survivors were identified from the SJLIFE cohort. Among these individuals, 562 refused participation in the intervention (a six-month randomized controlled trial of melatonin, identifier NCT01700959), and 1875 did not meet inclusion criteria upon an initial medical record review and/or phone screening. The remaining 911 survivors completed a study visit on the SJCRH campus that included baseline eligibility assessments. Of those who were eligible and randomized to the intervention (n=580), 477 survivors completed baseline self-reported sleep measures and at least three days of actigraphy and, thus, constituted the current study sample (supplemental Figure 1). All participants provided written informed consent, and the study was approved by the Institutional Review Board at SJCRH (00002731).

### Procedures

All study assessments (sleep measures, neurocognitive assessments, fatigue, psychological health surveys) were collected over two consecutive weeks. Most participants (85.7%) completed self-reported sleep and actigraphic sleep assessments within the same week. During the baseline campus visit, demographic, neurocognitive, sleep, fatigue, and psychological health measures were completed. Neurocognitive assessment entailed a two-hour battery of tests administered by a licensed psychological examiner under the general supervision of a board-certified neuropsychologist. Participants were provided with an actigraphy device and instructed to wear the device in their home sleep environment for five consecutive nights.

### Measures

#### Self-reported sleep.

The Pittsburgh Sleep Quality Index (PSQI)[25], a reliable and valid measure, was used to assess self-reported sleep over the previous 30 days. The PSQI measures sleep quality and quantity over the previous month and is comprised of 19 items scored on a 4-point Likert scale (0=not at all during the past month to 3=three or more times a week). Three components of the PSQI were analyzed as individual self-reported outcomes: sleep duration, sleep onset latency (SOL), and sleep efficiency (SE). Sleep duration was assessed via the question, “during the past month, how many hours of actual sleep did you get a night?” SOL was assessed via a PSQI question, “during the past month, how long in minutes has it taken you to fall asleep each night?” SE was calculated as the ratio of total sleep time compared to total time spent in bed.

#### Sleep quality.

Sleep quality was assessed via the overall composite score from the PSQI measure[25]. Possible scores range from 0 to 21, with higher scores indicating poorer sleep quality. A clinical cut-off score of > 5 was used to indicate poor sleep quality, scores ≤ 5 indicated good sleep quality.

#### Actigraphy-assessed sleep.

The Motionlogger Sleep Watch (Ambulatory Monitoring Inc, Ardsley, NY) was provided to each participant for sleep assessment. Participants were instructed to push an event button on the watch to designate bed time and wake time. Actigraphy assesses movement exceeding the zero-crossing mode at 30-second epochs and measures the number of minutes below threshold movement for sleep duration, number of minutes in bed until decreased movement onset for SOL, and number of minutes maintained at decreased movement over number of minutes in bed for SE. Actigraphy software was used to compute sleep characteristics. Due to the variability in the data (weekdays and weekends) and the variable number of days participants wore actigraphy, data for actigraphic sleep measures were aggregated across the number of collection days using median values.

#### Demographic and survivorship characteristics.

Demographic and survivorship-related characteristics included sex, race/ethnicity, age at time of study participation, age at diagnosis, chemotherapy (yes/no), radiation exposure (cranial, non-cranial, none) and cancer diagnosis (leukemia, central nervous system [CNS] tumor, non-CNS solid tumor, Hodgkin lymphoma, non-Hodgkin lymphoma, and Ewing/Osteosarcoma).

#### Psychological health.

Anxiety and depression were measured using subscales of the Brief Symptom Inventory 18 (BSI-18)[26]. Using sex-specific normative data, survivors with a T-score ≥63 (90^th^ percentile) on either subscale indicated clinically significant levels of anxiety and/or depression.

#### Fatigue.

The Functional Assessment of Chronic Illness Therapy Fatigue scale[27] (FACIT-Fatigue) assessed the intensity and functional influences of fatigue. The FACIT-Fatigue consists of 13 self-report items with possible scores ranging from 0 to 52. Lower scores indicate increased fatigue and a cut-off value of <30 was used to indicate severe fatigue associated with functional impairment[28].

#### Neurocognitive impairment.

Neurocognitive performance-based measures in the domains of attention, memory, and executive function were obtained from the Conner’s Continuous Performance Test II (CPT variability [sustained attention], omissions [inattention][29]; California Verbal Learning Test-II CVLT-II; Total [verbal learning][30]; Trail Making Test (Part B [cognitive flexibility])[31]; Controlled Oral Word Association Test (COWA; FAS [verbal fluency])[32]. Scores from all measures were converted into age-adjusted Z-scores (M=0, SD=1.0) and dichotomized as impaired or not impaired. Impairment was defined as a Z-score ≤ −1.5 on any one measure.

### Data Analysis

To measure agreement between self-report and actigraphy, Bland-Altman plots examined differences between self-reported and actigraphy-assessed measures of sleep (sleep duration, sleep onset latency [SOL], and sleep efficiency [SE]) plotted against the average of the two measures. Self-reported and actigraphic sleep measures were also converted into ordinal categories for calculation of weighted kappa coefficients. To assess the correlations between self-reported and actigraphic sleep measures, Pearson correlation coefficients were computed for each sleep variable. In addition to assessing agreement and associations between self-report and actigraphy measures, we examined late effects associated with bias (i.e. measurement difference), defined as the degree to which self-reported measures over or underestimated actigraphy assessments[10]. Mann-Whitney tests compared the absolute difference of measures between self-report and actigraphy for each of the sleep variables (duration, SOL, SE) among dichotomous late effect variables (anxiety, depression, poor sleep quality, fatigue and neurocognitive impairment). Absolute difference between sleep measures was examined as the outcome variable to allow for assessment of concordance due to the sample consisting of participants who reported higher or lower sleep on the PSQI compared to actigraphic sleep measures.

Multivariable general linear models for sleep duration, SOL, and SE examined the associations between survivor late effects (anxiety, depression, poor sleep quality, fatigue and neurocognitive impairment) and measurement difference (continuous outcome) while adjusting for age, sex, race/ethnicity. Unstandardized beta estimates and 95% confidence intervals were reported. For general linear models, the absolute difference between measures of self-report and actigraphy-assessed sleep were reported for each sleep variable. To also account for directional differences between self-report and actigraphic measures, separate general linear models were computed to examine factors associated with measurement differences of each sleep variable (duration, SOL, SE) among those participants who reported higher or lower sleep on the PSQI relative to actigraphy. Directional difference scores were created by subtracting self-reported sleep variables from actigraphy-assessed sleep variables. We subtracted self-reported sleep from actigraphy for these designations (instead of vice-versa) to be able to assess directionality of reporting. “Lower self-reporters” were classified separately for each sleep variable (duration, SOL, and SE) and included the following designations: a lower self-reported sleep duration relative to actigraphy, or a lower self-reported sleep efficiency relative to actigraphy, or a higher self-reported SOL relative to actigraphy. “Higher self-reporters” were classified as participants who self-reported a higher sleep duration relative to actigraphy, or self-reported a higher SE relative to actigraphy, or self-reported a lower SOL relative to actigraphy. All analyses were determined a priori, with the exception of the assessment of directional differences. Directional differences were completed post-hoc to aid in the interpretation of our findings.

## Results

### Clinical Characteristics of Survivors

Characteristics of the participants are summarized in Table 1. Participating survivors were 53.5% female, 87.4% non-Hispanic white, a median (range) age of 34.3 (19.3–61.6) years old at the time of study participation, and median (range) 25.4 (10.9–49.3) years from diagnosis. The sample consisted of 42.6% leukemia survivors and 23.5% solid tumors (non-central nervous system); 25.5% of survivors received cranial radiation, and 30.6% non-cranial radiation.

### Sleep Characteristics of Survivors

Survivors wore actigraphs for a median of 5 nights, with a range of 3–11 nights. Largely, survivors (n=358, 75%) wore actigraphs for 5–6 nights, with 12.2% of survivors (n=58) wearing actigraphs for 3–4 nights, and 12.8% of survivors for more than 6 nights (Supplemental Table 2). On average, participants self-reported a sleep duration of 394 minutes (6 hours 34 minutes), compared to 433 minutes (7 hours and 13 minutes) assessed via actigraphy. The mean PSQI score for the sample was 7.5, with 65.8% of the sample (n=311) reporting poor sleep quality. The directional differences between self-report and actigraphy indicated that, overall, participants were more likely report less self-reported sleep relative to their actigraphy assessment. For example, 68.7% (n=327) of the sample were classified as low self-reporters for sleep duration, compared to 31.3% (n=149) high self-reporters for sleep duration, and one participant reported a habitual sleep duration that matched their median actigraphy value. The proportion of participants who were classified as higher or lower self-reporters are presented by survivor characteristics in Supplemental Tables 3a-c. A greater proportion of survivors with poor sleep quality were classified as lower self-reporters for sleep duration (χ^2^ = 66.5, p≤0.0001) compared to those without poor sleep quality. The proportion of survivors classified as higher self-reporters for sleep duration was greater among participants with an impairment in verbal fluency (χ^2^ = 5.7, p=0.02).

### Agreement and Associations between Self-Report and Actigraphy Measures of Sleep

**Figure 1.**
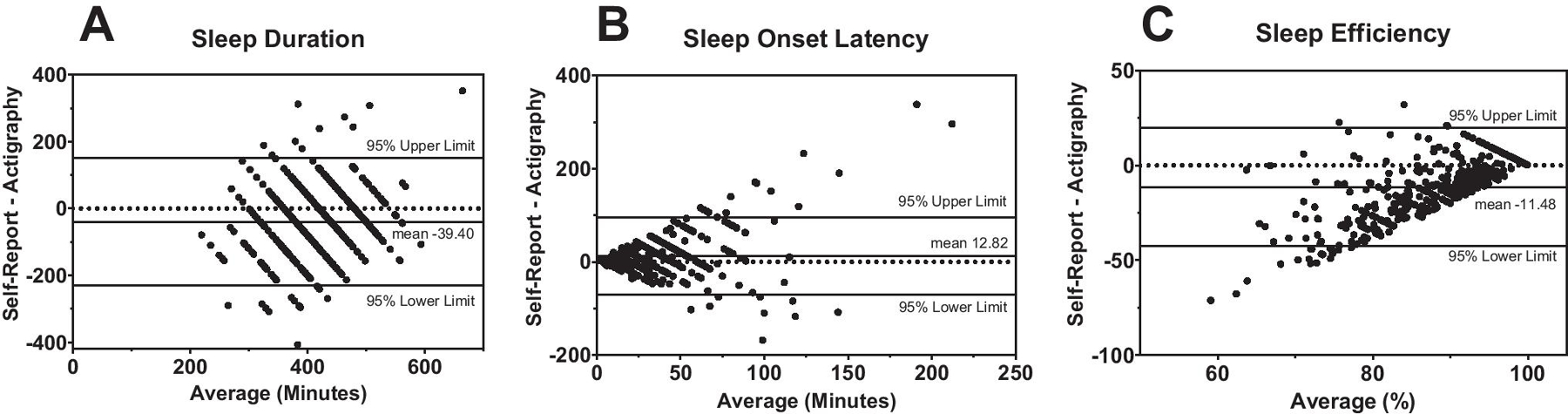
Bland-Altman Plots between Self-Reported and Actigraphy-Assessed Sleep Outcomes in a Sample of Adult Survivors of Childhood Cancer (n=477) Bland-Altman plots of sleep duration (A), sleep onset latency (B), and sleep efficiency (C) show the difference between self-reported sleep and actigraphy-assessed sleep (y-axis), plotted against the average value of the two measures (x-axis). The horizontal reference line (dotted) represents zero difference between self-reported and actigraphic measures. The solid line labeled mean represents the bias, reflecting the difference the mean values of the self-reported and actigraphic measures and the 95% upper and lower limits reflect the limits of agreement (mean 1.96 × SD). In Figure A., the mean of −39.40 reflects that self-reported sleep duration was underestimated relative to actigraphic sleep duration by approximately 39 minutes on average. In Figure B, self-reported sleep onset latency was overestimated relative to actigraphic SOL by an average of 13 minutes. In Figure C, self-reported sleep efficiency was underestimated by an average of 11% relative to actigraphic sleep efficiency.

Bland-Altman plots assessing agreement between self-reported and actigraphy measures of sleep (duration, sleep onset latency [SOL], and sleep efficiency [SE]) are presented in Figure 1. Lower and upper agreement limits are displayed for each plot and indicate clinically significant disagreement between measures. In addition, the plots show that as average sleep onset latency increased (Figure 1B) and average sleep efficiency (Figure 1C) decreased, the discrepancy between measures increased.

When sleep outcomes were compared categorically, weighted kappa coefficients for sleep duration and SOL indicated a slight to fair agreement between measures (*k_w_*=0.20, *k_w_*=0.22, respectively, p<0.0001), there was no agreement between measures of SE (*k_w_*=0.00, p=0.79) (Table 2). Correlations between actigraphy measures and self-reported sleep duration, SOL, and SE are summarized in Table 2. Correlations between self-report and actigraphy measures for SOL and sleep duration were small, albeit statistically significant (r=0.22, r=0.24, respectively; p<0.0001). There was not a statistically significant association between self-reported and actigraphy-measured SE (r=0.05, p=0.27).

### Univariate Associations: Absolute Measurement Differences

The following late effects variables were associated with statistically significant absolute differences between self-reported and actigraphic measures of sleep duration in univariate analyses: poor sleep quality (p<0.0001), fatigue (p=0.01), and an impairment in memory (p<0.001) (Supplemental Table 4a). The following variables were associated with statistically significant absolute differences between self-reported and actigraphic measures of sleep onset latency: poor sleep quality (p<0.0001), fatigue (p<0.01), anxiety (p<0.01) and depression (p<0.001) (Supplemental Table 4b). The following variables were associated with statistically significant absolute differences between self-reported and actigraphic measures of sleep efficiency: depression (p=0.03) and poor sleep quality (p<0.0001) (Supplemental Table 4c).

### Multivariable Associations: Absolute and Directional Measurement Differences

Table 3 summarizes the multivariable general linear models for absolute differences of self-reported vs. actigraphy measured sleep duration, sleep onset latency [SOL] and sleep efficiency [SE] and associations between late effects (poor sleep quality fatigue, anxiety, depression, impaired memory). Severe fatigue, versus not and poor sleep quality, versus not, were each associated with an approximate 26-minute higher absolute difference between self-reported and actigraphy-assessed sleep duration (*B*=26.6, p<0.001; *B*=26.8, p<0.01, respectively). Memory impairment accounted for a 44-minute higher absolute difference between self-report and actigraphy-assessed sleep duration (*B*=44.4, p<0.001), compared to those without a memory impairment. Depression, versus not, and poor sleep quality, versus not, were each statistically significantly associated with a 24.5 and 16.4 minute higher absolute discrepancy of self-reported and actigraphy-measured sleep onset latency (*B*=24.5, p<0.01 and *B*=16.4, p<0.0001, respectively). Poor sleep quality was associated with a 12% increase in the absolute difference between self-reported sleep efficiency and actigraphic measures (*B*=12.3, p<0.0001). When considering directional differences, poor sleep quality (vs. not) was consistently associated with a statistically significant increase in measurement differences for sleep duration (*B*=45.01, p<0.0001), SOL (*B*=18.6, p<0.01) and SE (*B*=13.2, p<0.0001) among lower self-reporters (Supplemental Table 5). Severe fatigue (vs not) and memory impairment (vs. no impairment) were associated with both an increase in measurement differences of sleep duration among lower self-reporters (*B*=22.0, p=0.07; *B*=31.4, p=0.04, respectively) and higher self-reporters (*B*=66.7, p<0.001; *B*=46.9, p=0.03, respectively) see Supplemental Tables 5-6.

## Discussion

In a large clinically-assessed cohort of adult survivors of childhood cancer with neurocognitive impairment and/or a prolonged SOL, we examined the concordance between self-reported sleep and actigraphy-assessed sleep in relation to psychological and neurocognitive late effects. We observed that poor sleep quality and common psychological and neurocognitive late effects were associated with increased discrepancies between self-reported and actigraphic sleep measures. These findings highlight the complexity of sleep measurement, particularly among medically complex patient populations and the need for future research to study sleep measurement among survivors.

Consistent with our results, a study of breast cancer survivors found significant disagreement between daily sleep diaries and actigraphy[11]. However, in breast cancer survivors[11], patient characteristics including insomnia and fatigue were not associated with variability in agreement between self-reported and actigraphic sleep measures, leading the authors to conclude that the lack of association may have been due to a small sample size (n=43). Several studies in non-cancer samples have identified the lack of concordance between self-reported sleep and actigraphy-assessed sleep as driven by factors such as poor sleep quality[13, 33], insomnia[10], mood disturbance[17], and cognitive impairment[33]. Collectively, our present findings as well as previous results highlight the importance of considering factors associated with measurement discordance when comparing self-report and actigraphy-assessed sleep in childhood cancer survivors. In our adjusted models, depression, fatigue, poor sleep quality, and memory impairment were independently associated with greater discrepancies between self-reported and actigraphic measures of sleep. These findings are meaningful because the factors associated with measurement discordance in our sample are very common late effects of childhood cancer. Moreover, our overall findings of weak agreement between self-reported and actigraphy-assessed sleep may be driven by the high prevalence of survivors with poor sleep quality, severe fatigue, and neurocognitive impairment in our sample. Thus, discrepancies between self-report and actigraphy sleep measures may be more pronounced among childhood cancer survivors due to the prevalence of these late effects as compared to non-cancer populations.

Findings from the present study should not be interpreted as suggesting either self-report or actigraphy are unreliable measures of sleep. Rather, our data (and that of others) suggest that these measures are different from one another. Due to the lack of polysomnographic (PSG) data, the extent to which survivors underestimated their self-reported sleep or the extent to which actigraphy overestimated sleep as assessed via PSG is unknown; however, PSG also has limitations. While actigraphy has been strongly correlated to PSG in healthy individuals[34], actigraphy was found to overestimate sleep duration by an average of twenty-two minutes compared to polysomnography in a chronically ill patient population[18]. The validity of actigraphy for sleep onset latency in individuals with a sleep disorder may also be less reliable. There may also be less consistency in actigraphic measurement of sleep onset latency (when validated by PSG or daily sleep diaries) compared to actigraphic measures of total sleep time and wake after sleep onset as described by a review of actigraphy validation[35]. These findings underscore the complexity of different sleep measures and highlights that this complexity should be considered when studying patient populations experiencing medical comorbidities and sleep disturbances.

Further complicating measurement, the etiology of sleep disorders, such as hypersomnia/narcolepsy, sleep-disordered breathing, and insomnia may be different in cancer survivors due to disease specific factors (e.g. tumor location within the brain) or treatment exposures. For instance, craniopharyngioma survivors have a high rate of excessive daytime sleepiness and meet criteria for secondary narcolepsy and hypersomnia[36], related to the degree of hypothalamic involvement of their tumor[5]. Survivors who receive radiation therapy have an increased risk of obstructive sleep apnea[37], which may be related to thoracic radiation that has structurally changed survivors’ upper airway systems. Insomnia, a multifactorial sleep disorder, may present differently in cancer survivors due to several perpetuating factors that can be associated with survivorship, psychological distress, fatigue, pain, hormonal disruptions, and chronic health conditions associated with treatment exposures[38–40, 4]. These differences in etiology and/or presentation of clinical sleep disorders in cancer survivors are understudied and may increase the challenges of sleep measurement among medically complex patients. Thus, it is imperative that further investigation of sleep measures be conducted among cancer survivors. Such research is needed to understand the variability across sleep measures in this unique population. While the determination of an “optimal” measure of sleep would likely differ across studies, this decision involves the consideration of feasibility, study aims, sleep time frame of interest and sleep disturbance of interest. Thus, when selecting sleep measures, it is essential oncology researchers be equipped with an understanding that several types of sleep measures may yield different findings. Including multiple measures of sleep, when possible, may also help improve our understanding of sleep among cancer survivors.

Results of our study should be considered within the context of several limitations. Participants were instructed to wear actigraphy devices for five nights (three weekday and two weekend); however, there was some variability in this as actigraphy data ranged from three to 11 nights. In addition, despite our large sample, survivors were specifically recruited for a pharmaceutical intervention targeting sleep and neurocognition. Due to this, our sample had either a prolonged sleep onset latency (SOL) and/or a neurocognitive impairment. Therefore, the prevalence of poor sleep quality and prolonged self-reported SOL may be overestimations of the prevalence of sleep disturbances among all childhood cancer survivors. Irrespective of this, the discordance found between actigraphy and self-reported sleep measures highlight the importance of using multiple measures in patient populations, particularly those with poor sleep quality. When studying absolute and directional differences, our use of the PSQI total score as a predictor of discrepancy between PSQI-assessed and actigraphy-assessed sleep may have introduced some measurement bias into our findings that we are unable to fully ascertain. However, the dependent variable was defined as the absolute difference between measures (actigraphy and PSQI) and not a single item (e.g. sleep duration) or a component measure of the PSQI. The PSQI broadly assesses dimensions of sleep over the previous month. Furthermore, it does not allow individuals to account for nightly differences in their sleep, which could be captured by a sleep diary or actigraphy measures. Thus, we acknowledge that examining the concordance between a daily diary and actigraphy versus the concordance between the PSQI and actigraphy, could potentially yield different results, as comparing a general estimate of sleep to actigraphy has the potential to heighten the discrepancies between these measures. Moreover, the concordance between actigraphy and another self-reported scale that is validated to measure sleep over a briefer time span (e.g. the PROMIS- Sleep Disturbance) or utilizing more than five days of actigraphy could potentially yield different results. While several studies reporting discordance among sleep measures have often relied on comparing sleep measures across different time frames [10, 17, 14], it is important to note that discordance has also been reported across similar measurement time frames such as comparing nightly actigraphy and daily sleep diaries over a short time frame (ten days) [15] and over long periods of time (five weeks) [11]. Thus, the time frame difference between measures may not have been driving the discordance, despite this limitation.

Despite these limitations, the comparison of PSQI-assessed sleep and actigraphy-assessed sleep has practical value, as it is not uncommon for researchers to utilize both validated questionnaires and actigraphy in studies. If both measures are utilized, and researchers expect strong concordance or employ actigraphy as a means to “validate” self-reported sleep, this can be problematic. Furthermore, beyond quantifying the distinctions between these measurement approaches, we also found that specific late effects of childhood cancer were associated with greater differences between self-reported and actigraphic sleep measures. Discordance between sleep measures can vary by patient population and may be more pronounced among cancer survivors who experience neurocognitive and/or psychological late effects.

Adult survivors of childhood cancer are at risk for numerous late effects including fatigue, poor sleep quality, depression, and neurocognitive impairment that may increase the magnitude of disagreement between self-reported and actigraphic measures of sleep. The discrepancies observed in the current study raise questions identified in previous research, such as whether self-reported sleep and actigraphy may be reflecting different aspects of sleep[17]. Incorporating actigraphy into survivorship research can provide additional sleep data, but it should not be done as a means of “validating” self-reported sleep, as our findings show substantial differences between these measures. Determining sleep measurement approaches in survivorship research should be done with thoughtful consideration of the survivor population and sleep parameters of interest, as well as within the context of considering the potential presence of late effects and how these may relate to differences found between self-reported and actigraphy-assessed sleep.

## Supplementary Material

Supplemental Material

## Figures and Tables

**Table 1.** Characteristics of Study Sample (n=477) from SJLIFE Cohort

Survivor Characteristics	Median (range)
Age at evaluation (years)	34.28 (19.26–61.6))
Age at diagnosis (years)	8.00 (<1 – 20.00)
Time since diagnosis (years)	25.43 (10.94–49.32)
**Sex**	**N (%)**
Male	222 (46.5%)
Female	255 (53.5%)
**Race/Ethnicity**	
Non-Hispanic, white	417 (87.4%)
**Diagnosis group**	
Leukemia	203 (42.6%)
Central nervous system (CNS) tumor	41 (8.6%)
Non-CNS solid tumor	112 (23.5%)
Hodgkin lymphoma	53 (11.1%)
Non-Hodgkin lymphoma	24 (5.0%)
Ewing/Osteosarcoma	36 (7.6%)
**Chemotherapy**	
Yes	417 (87.4%)
No	60 (12.6%)
**Radiation**	
Cranial	119 (25.0%)
Non-Cranial	146 (30.6%)
None	212 (44.4%)
**Psychological Health** ^†^	
Depression (missing = 38)	27 (6.2%)
Anxiety (missing = 25)	25 (5.7%)
**Fatigue** ^‡^	
Severe Fatigue	61 (13.7%)
None to Moderate	385 (86.3%)
**Poor Sleep Quality** ^§^	
Yes	311 (65.8%)
No	162 (34.3%)
**Neurocognitive Impairment** ^¶^	
*Memory*	
Verbal Learning	40 (8.4%)
*Attention*	
Sustained Attention	78 (16.4%)
Inattention	60 (12.6%)
*Executive Function*	
Cognitive Flexibility	68 (14.3%)
Verbal Fluency	42 (8.8%)

† Anxiety and depression were defined as a T-score ≥63 on the Brief Symptom Inventory-18 measure for the depression or anxiety subscale

‡ Severe fatigue was defined as a score >30 on the FACIT Fatigue measure

§ Poor sleep quality was defined as a score > 5 on the Pittsburgh Sleep Quality Index

¶ Neurocognitive impairment defined as z-score ≤ 1.5 standard deviations below the mean

**Table 2.** Agreement between Categorical Designations (Kappa Coefficient) and Continuous Measures (Correlations) of Self-Reported and Actigraphy-Assessed Sleep Outcomes (n=477)

Sleep Outcome	Weighted Kappa^†^ (95% CI)		P-Value
Sleep duration^‡^	0.1951 (0.1315 – 0.2587)		0.0001
Sleep onset latency^§^	0.2186 (0.0339 – 0.1522)		0.0001
Sleep efficiency^¶^	0.0054 (−0.0333 – 0.0440)		0.79
**Sleep Outcome**	**Actigraphy** **Mean (SD)**	**Self-Report** **Mean (SD)**	**Pearson’s R**
Sleep duration (minutes)	433.41 (72.14)	394.09 (85.17)	0.24*
Sleep onset latency (minutes)	26.55 (27.11)	39.35 (39.05)	0.27*
Sleep efficiency (%)	94.17% (5.79)	82.76% (15.08)	0.05

† Weighted Kappa coefficients reported due to ordinal categories

‡ Sleep duration categories: <360 minutes; 360–419 minutes; 420–479 minutes; 480–540 minutes; >540 minutes

§ Sleep onset latency categories: <15 minutes; 15–20 minutes; 21–30 minutes; >30 minutes

¶ Sleep efficiency categories: <80%; 80–84%; 85–90%; >90%

* P < 0.05 for Pearson’s R test

**Table 3.** General Linear Models Examining the Associations between Survivor Late Effects and Absolute Differences Between Self-Reported and Actigraphic Sleep Measures in Sample from SJLIFE (n=405)

	Absolute Difference between Self-Reported and Actigraphic Measure							
	Sleep Duration (minutes)			Sleep Onset Latency (minutes)			Sleep Efficiency (%)	
**Survivor Late Effects**	*Β* (95% CI)	*P-value*		*Β* (95% CI)	*P-value*		*Β* (95% CI)	*P-value*
Anxiety^†^	*−9.39* (−42.48 – 23.69)	0.58		*4.23* (−13.55 – 22.01)	0.64		*−1.20* (−7.06 – 4.66)	0.69
Depression^†^	*5.44* (−27.33 – 38.21)	0.74		*24.49* (6.87 – 42.11)	0.01*		*2.08* (−3.73 – 7.89)	0.48
Severe fatigue^‡^	*26.84* (6.66 – 47.02)	0.01*		0.69 (−10.16 – 11.54)	0.90		*−0.39* (−3.97 – 3.18)	0.83
Poor sleep quality^§^	*26.58* (12.59 – 40.57)	0.001*		16.42 (8.90 – 23.94)	0.0001*		*12.34* (9.86 – 14.82)	0.0001*
Memory impairment^¶^	*44.36* (19.81 – 68.90)	0.001*		*−4.91* (−18.11 – 8.28)	0.46		*0.03* (−4.31 – 4.38)	0.99
Inattention impairment^¶^	*−3.63* (−25.24 – 17.99)	0.74		*4.09* (−7.54 – 15.71)	0.49		*−0.32* (−4.15 – 3.51)	0.87
Sustained attention impairment^¶^	*6.67* (−12.84 – 26.19)	0.50		*−0.22* (−10.71 – 10.28)	0.97		*2.53* (−0.93 – 5.99)	0.15
Cognitive flexibility impairment^¶^	*−1.40* (−21.81 – 19.01)	0.89		*2.12* (−8.85 – 13.10)	0.70		*1.12* (−2.50 – 4.73)	0.54
Verbal fluency impairment^¶^	*−17.56* (−41.21 – 6.10)	0.15		*1.97* (−10.74 – 14.69)	0.76		*−0.08* (−4.27 – 4.11)	0.97
**Demographic Covariates**								
Age at evaluation (per one year)	*−0.07* (−0.81 – 0.67)	0.85		*0.03* (−0.37 – 0.42)	0.90		*0.00* (−0.13 – 0.13)	0.98
Sex^††^	*−2.30* (−15.43 – 10.84)	0.73		*1.72* (−5.34 – 8.78)	0.63		*1.27* (−1.05 – 3.60)	0.28
Race/ethnicity^‡‡^	*−17.61* (−26.83 – 1.61)	0.07		*3.40* (−6.95 – 13.74)	0.52		*−1.85* (−5.25 – 1.56)	0.29

† Anxiety and depression were defined as a T-score ≥63 on the Brief Symptom Inventory-18 measure for the depression or anxiety subscale, reference group = no anxiety or depression

‡ Severe fatigue was defined as a score >30 on the FACIT Fatigue measure, reference group ≤ severe fatigue

§ Poor sleep quality was defined as a score >5 on the Pittsburgh Sleep Quality Index, reference group = normal sleep quality

¶ Neurocognitive impairment defined as z-score ≤ 1.5 standard deviations below the mean, reference group= no impairment

†† Females, reference group = males

‡‡ “Other”, reference group = non-Hispanic, white

## Data Availability

Research data are not shared.
