# Supplementary material for: Concordance between self-reported sleep and actigraphy-assessed sleep in adult survivors of childhood cancer: the impact of psychological and neurocognitive late effects
Source: Support Care Cancer. Author manuscript; Available in PMC 2023 Feb 1. (PMC8732302; doi:10.1007/s00520-021-06498-x)
Supplement: Supplemental Material [file NIHMS1740765-supplement-Supplemental_Material.docx]

**Supplemental File**

Supplemental Figure 1. Study Flow Diagram

Did not complete baseline actigraphy N=79

< 3 days of actigraphy N=4

Did not complete baseline sleep survey N=6

Started study measures > two weeks apart N=14

Participants, completed ≥ three days of baseline actigraphy and self-reported sleep measures

N=477

Potentially eligible patients identified

N=4298

Ineligible N=950

Potentially eligible for intervention recruitment

N=3348

Active refusals N=562

Ineligible N=1875

Participants, baseline tested

N=911

Ineligible after baseline screening N=298

Withdrew before randomization N=33

Participants, randomized to intervention

N=580

Supplemental Table 1. Intervention Study Inclusion and Exclusion Criteria for Sleep and Cognition Intervention

| Inclusion Criteria | Exclusion Criteria^†^ |
| --- | --- |
| 1) Previously treated at SJCRH and a participant in SJLIFE  2) 10 or more years from diagnosis  3) FSIQ score > 79  4) > 18 years of age  5) Able to speak and understand the English language  6) Self-reported sleep onset latency ≥30 minutes per night at least one time per week during the previous month and/or a neurocognitive impairment of <0.3 SD below the mean | 1) Known allergy to melatonin or any ingredients of the study product or placebo  2) Currently taking Melatonin  3) Known sleep apnea or other medically treated sleep disorder  4) Known diabetes mellitus – insulin treated  5) Uncontrolled seizure disorder in the past 12 months  6) Reported current illicit drug or alcohol abuse or dependence  7) Reported current major psychiatric illness (i.e. schizophrenia, bipolar disorder)  8) Current treatment with:  a. Benzodiazepines or other CNS depressants  b. Fluvoxamine  c. Anticoagulants (e.g. Coumadin)  d. Immunosuppressant or corticosteroids  e. Nifedipine  9) Employed in a position that requires night work (i.e. 10pm to 6am)  10) Females who are pregnant or lactating/nursing  11) History of neurologic event unrelated to cancer or its treatment  12) Sensory impairment (vision, hearing) that prohibits completion of neurocognitive examination |
|  |  |
|  |  |
|  |  |
|  |  |
|  |  |
|  |  |

SJCRH = St. Jude Children’s Research Hospital; SJLIFE = St. Jude Lifetime Cohort Study; FSIQ = Full Scale Intelligence Quotient

Supplemental Table 2. Number of Consecutive Days Participants Completed Actigraphy

| **Number of Days**^†^ | **N (%)** |
| --- | --- |
| Three | 10 (2.1) |
| Four | 48 (10.1) |
| Five | 282 (59.1) |
| Six | 76 (15.9) |
| Seven | 50 (10.5) |
| Eight | 7 (1.5) |
| Nine | 2 (0.4) |
| Ten | 1 (0.2) |
| Eleven | 1 (0.2) |

^†^participants were instructed to wear the actigraphs for five nights and return the device via mail; however some individuals completed additional nights before returning the device to the research team.

Supplemental Table 3a. The Proportion of Higher Self-Reporters and Lower Self-Reporters of Sleep Duration by Survivor Characteristics (n=477)

| **Characteristic** | **Higher Self-Reporter**^†^  **N (%)** | **Lower Self-Reporter**^‡^  **N (%)** | **Chi-Square** | **P-Value** |
| --- | --- | --- | --- | --- |
| **Sex** |  |  | 3.79 | 0.05 |
| Female | 70 (27.5%) | 185 (72.6%) |  |  |
| Male | 79 (35.8%) | 142 (64.3%) |  |  |
| **Race/ethnicity** |  |  | 0.9186 | 0.34 |
| Non-Hispanic, white | 127 (30.5%) | 289 (69.5%) |  |  |
| Other | 22 (36.7%) | 38 (63.3%) |  |  |
| **Depression** |  |  | 1.3101 | 0.25 |
| Yes | 6 (22.2%) | 21 (77.8%) |  |  |
| No | 135 (32.9%) | 276 (67.2%) |  |  |
| **Anxiety** |  |  | 0.8151 | 0.37 |
| Yes | 6 (24.0%) | 19 (76.0%) |  |  |
| No | 135 (32.7%) | 278 (67.3%) |  |  |
| **Fatigue** |  |  | 0.6119 | 0.43 |
| Severe | 16 (26.7%) | 44 (73.3%) |  |  |
| < Severe | 122 (31.7%) | 263 (68.3%) |  |  |
| **Poor Sleep Quality** |  |  | 66.5351 | <0.0001* |
| Yes | 57 (18.4%) | 253 (81.6%) |  |  |
| No | 89 (59.9%) | 73 (45.1%) |  |  |
| **Memory Impairment** |  |  | 0.0291 | 0.86 |
| Yes | 13 (32.5%) | 27 (67.5%) |  |  |
| No | 136 (31.2%) | 300 (68.8%) |  |  |
| **Sustained Attention Impairment** |  |  | 0.1430 | 0.71 |
| Yes | 23 (29.5%) | 55 (70.5%) |  |  |
| No | 126 (31.7) | 398 (83.6) |  |  |
| **Inattention Impairment** |  |  | 0.1317 | 0.72 |
| Yes | 20 (33.3%) | 40 (66.7%) |  |  |
| No | 129 (31.0%) | 287 (69.0%) |  |  |
| **Executive Function Impairment** |  |  | 0.0852 | 0.77 |
| Yes | 22 (32.8%) | 45 (67.2%) |  |  |
| No | 127 (31.1%) | 282 (69.0%) |  |  |
| **Verbal Fluency Impairment** |  |  | 5.7030 | 0.02* |
| Yes | 20 (47.6%) | 22 (52.4%) |  |  |
| No | 129 (29.7%) | 305 (70.3%) |  |  |

^†^Higher self-reporter refers self-reporting a greater sleep duration compared to the actigraphy measures

^‡^Lower self-reporter refers to self-reporting a decreased sleep duration compared to the actigraphy measure

* P < 0.05

Supplemental Table 3b. The Proportion of Higher Self-Reporters and Lower Self-Reporters of Sleep Onset Latency by Survivor Characteristics (n=477)

| **Characteristic** | **Higher Self-Reporter**^†^  **N (%)** | **Lower Self-Reporter**^‡^  **N (%)** | **Chi-Square** | **P-Value** |
| --- | --- | --- | --- | --- |
| **Sex** |  |  | 2.5369 | 0.11 |
| Female | 81 (31.8%) | 174 (68.2%) |  |  |
| Male | 86 (38.7%) | 136 (62.3%) |  |  |
| **Race/ethnicity** |  |  | 6.7775 | 0.001* |
| Non-Hispanic, white | 137 (32.9%) | 280 (67.2%) |  |  |
| Other | 30 (50.0%) | 30 (50.0%) |  |  |
| **Depression** |  |  | 2.0211 | 0.16 |
| Yes | 6 (22.2%) | 21 (77.8%) |  |  |
| No | 147 (35.7%) | 265 (64.3%) |  |  |
| **Anxiety** |  |  | 1.3750 | 0.24 |
| Yes | 6 (24.0%) | 19 (76.0%) |  |  |
| No | 147 (35.5%) | 267 (64.5%) |  |  |
| **Fatigue** |  |  | 1.8651 | 0.17 |
| Severe | 17 (27.8%) | 44 (72.1%) |  |  |
| < Severe | 142 (36.9%) | 243 (63.1%) |  |  |
| **Poor Sleep Quality** |  |  | 32.1040 | <0.0001* |
| Yes | 80 (48.2%) | 231 (74.2%) |  |  |
| No | 84 (51.9%) | 78 (25.7%) |  |  |
| **Memory Impairment** |  |  | 2.9933 | 0.08 |
| Yes | 19 (47.5%) | 21 (52.5%) |  |  |
| No | 148 (33.9%) | 289 (66.1%) |  |  |
| **Sustained Attention Impairment** |  |  | 0.0322 | 0.856 |
| Yes | 28 (35.9%) | 50 (64.1%) |  |  |
| No | 139 (34.8%) | 260 (65.2%) |  |  |
| **Inattention Impairment** |  |  | 2.0895 | 0.15 |
| Yes | 26 (43.3%) | 34 (56.7%) |  |  |
| No | 141 (33.8%) | 276 (66.2%) |  |  |
| **Executive Function Impairment** |  |  | 3.8999 | 0.05* |
| Yes | 31 (45.6%) | 37 (54.4%) |  |  |
| No | 136 (33.3%) | 273 (66.8%) |  |  |
| **Verbal Fluency Impairment** |  |  | 0.6047 | 0.45 |
| Yes | 17 (40.5%) | 25 (59.5%) |  |  |
| No | 150 (34.5%) | 285 (65.5%) |  |  |

^†^higher self-reporter refers self-reporting a greater sleep duration compared to the actigraphy measures

^‡^lower self-reporter refers to self-reporting a decreased sleep duration compared to the actigraphy measure

* P < 0.05

Supplemental Table 3c. The Proportion of Higher Self-Reporters and Lower Self-Reporters of Sleep Efficiency by Survivor Characteristics (n=477)

| **Characteristic** | **Higher Self-Reporter**^†^  **N (%)** | **Lower Self-Reporter**^‡^  **N (%)** | **Chi-Square** | **P-Value** |
| --- | --- | --- | --- | --- |
| **Sex** |  |  | 11.5662 | 0.001* |
| Female | 53 (20.8%) | 202 (79.2%) |  |  |
| Male | 77 (34.7%) | 145 (65.3%) |  |  |
| **Race/ethnicity** |  |  | 0.5320 | 0.47 |
| Non-Hispanic, white | 116 (27.8%) | 301 (72.2%) |  |  |
| Other | 14 (23.3%) | 46 (76.7%) |  |  |
| **Depression** |  |  | 0.4792 | 0.49 |
| Yes | 6 (22.2%) | 21 (77.8%) |  |  |
| No | 117 (28.4%) | 295 (71.6%) |  |  |
| **Anxiety** |  |  | 0.2084 | 0.65 |
| Yes | 8 (32.0%) | 17 (68.0%) |  |  |
| No | 115 (27.8%) | 299 (72.2%) |  |  |
| **Fatigue** |  |  | 0.7577 | 0.38 |
| Severe | 14 (23.0%) | 47 (77.1%) |  |  |
| < Severe | 109 (28.3%) | 276 (71.7%) |  |  |
| **Poor Sleep Quality** |  |  | 43.7499 | <0.0001* |
| Yes | 55 (17.7%) | 256 (82.3%) |  |  |
| No | 75 (46.3%) | 87 (53.7%) |  |  |
| **Memory Impairment** |  |  | 0.0013 | 0.97 |
| Yes | 11 (27.5%) | 29 (72.5%) |  |  |
| No | 119 (27.2%) | 318 (72.8%) |  |  |
| **Sustained Attention Impairment** |  |  | 0.1223 | 0.73 |
| Yes | 20 (25.6%) | 58 (74.4%) |  |  |
| No | 110 (27.6%) | 289 (72.4%) |  |  |
| **Inattention Impairment** |  |  | 0.2611 | 0.61 |
| Yes | 18 (30.0%) | 42 (70.0%) |  |  |
| No | 112 (26.9%) | 305 (73.1%) |  |  |
| **Executive Function Impairment** |  |  | 0.0245 | 0.88 |
| Yes | 18 (26.5%) | 50 (73.5%) |  |  |
| No | 112 (27.4%) | 297 (72.6%) |  |  |
| **Verbal Fluency Impairment** |  |  | 7.5134 | 0.01* |
| Yes | 19 (45.2%) | 23 (54.8%) |  |  |
| No | 111 (25.2%) | 324 (74.5%) |  |  |

^†^higher self-reporter refers self-reporting a greater sleep duration compared to the actigraphy measures

^‡^lower self-reporter refers to self-reporting a decreased sleep duration compared to the actigraphy measure

* P < 0.05

Supplemental Table 4a. Absolute Difference in Measures of Sleep Duration by Categorical Predictors

|  | **N** | **Median (range); minutes** | **95% CL** | **Z** | **P-value** |
| --- | --- | --- | --- | --- | --- |
| **Female** | 255 | 66.5 (406) | 60 to 72 | -1.0545 | 0.29 |
| **Male** | 222 | 58 (352) | 50 to 69 |  |  |
|  |  |  |  |  |  |
| **Non-Hispanic White** | 417 | 65 (407) | 58 to 70 | -0.7112 | 0.48 |
| **Other** | 60 | 60 (306) | 43 to 79 |  |  |
|  |  |  |  |  |  |
| **Psychological Health** | | | | | |
| **Anxiety** | 25 | 69 (288.5) | 51 to 131 | 0.8490 | 0.40 |
| **No Anxiety** | 414 | 64 (407) | 56 to 71 |  |  |
|  |  |  |  |  |  |
| **Depression** | 27 | 78.5 (276) | 57 to 131 | 1.3936 | 0.16 |
| **No Depression** | 412 | 63 (407) | 53 to 69 |  |  |
| **Sleep Quality** | | | | | |
| **Poor Sleep Quality** | 311 | 72.5 (407) | 66.5 to 83.0 | -5.1373 | <0.0001* |
| **Good Sleep Quality** | 162 | 46 (351) | 41.5 to 53 |  |  |
|  |  |  |  |  |  |
| **Fatigue** | | | | | |
| **Severe Fatigue** | 61 | 79 (407) | 66.5 to 116 | 2.5987 | 0.001* |
| **< Severe Fatigue** | 385 | 62 (351) | 52.5 to 68 |  |  |
|  |  |  |  |  |  |
| **Neurocognitive Impairment** | | | | | |
| **Memory Domain** | | | | | |
| **Memory Impairment** | 40 | 83.5 (331) | 66 to 156 | 3.5246 | <0.001* |
| **No Impairment** | 437 | 61 (407) | 52 to 69 |  |  |
|  | | | | | |
| **Inattention Impairment** | 60 | 58 (306.5) | 41 to 84 | -0.4643 | 0.64 |
| **No Impairment** | 417 | 65 (407) | 58 to 70 |  |  |
|  |  |  |  |  |  |
| **Sustained Attention Impairment** | 78 | 63 (307) | 48 to 86 | 0.1563 | 0.88 |
| **No Impairment** | 399 | 64 (407) | 54 to 69 |  |  |
| **Executive Functioning Domain** | | | | | |
| **Cognitive Flexibility Impairment** | 68 | 64.5 (352) | 51 to 87.5 | 0.0570 | 0.96 |
| **No Impairment** | 409 | 63 (406) | 53 to 69 |  |  |
|  |  |  |  |  |  |
| **Verbal Fluency Impairment** | 42 | 62 (175) | 40 to 87.5 | -0.2169 | 0.83 |
| **No Impairment** | 435 | 64 (407) | 56 to 69 |  |  |

P-values are based on Mann-Whitney tests

* P < 0.05

Supplemental Table 4b. Absolute Difference in Measures of Sleep Onset Latency by Categorical Predictors

|  | **N** | **Median (range); minutes** | **95% CL** | **Z** | **P-value** |
| --- | --- | --- | --- | --- | --- |
| **Female** | 255 | 17 (338) | 15 to 20 | -1.2761 | 0.20 |
| **Male** | 222 | 15 (168) | 12 to 19 |  |  |
|  |  |  |  |  |  |
| **Non-Hispanic White** | 416 | 16.75 (338) | 14 to 18 | 0.5885 | 0.56 |
| **Other** | 60 | 18.5 (232.5)_ | 9 to 27 |  |  |
|  |  |  |  |  |  |
| **Psychological Health** | | | | | |
| **Anxiety** | 25 | 37 (228)_ | 14 to 51 | 2.9580 | 0.01* |
| **No Anxiety** | 413 | 16.5 (338) | 14 to 19 |  |  |
|  |  |  |  |  |  |
| **Depression** | 27 | 35 (334.5) | 20 to 51 | 3.3384 | 0.001* |
| **No Depression** | 411 | 16 (296) | 14 to 18 |  |  |
| **Sleep Quality** | | | | | |
| **Poor Sleep Quality** | 311 | 20 (338) | 18 to 23 | -7.1019 | <0.0001* |
| **Good Sleep Quality** | 162 | 9 (102.5) | 6 to 11 |  |  |
|  |  |  |  |  |  |
| **Fatigue** | | | | | |
| **Severe Fatigue** | 61 | 22 (293.5) | 17 to 30.5 | 3.1512 | 0.01* |
| **< Severe Fatigue** | 384 | 16 (338) | 13 to 18 |  |  |
|  |  |  |  |  |  |
| **Neurocognitive Impairment** | | | | | |
| **Memory Domain** | | | | | |
| **Memory Impairment** | 39 | 16 (118) | 8 to 25 | -0.5359 | 0.59 |
| **No Impairment** | 437 | 17 (338) | 14.5 to 19 |  |  |
|  | | | | | |
| **Inattention Impairment** | 59 | 19 (189.5) | 9.5 to 30 | 0.7566 | 0.45 |
| **No Impairment** | 417 | 17 (338) | 14 to 18 |  |  |
|  |  |  |  |  |  |
| **Sustained Attention Impairment** | 77 | 17 (190.5) | 10 to 24 | 0.3494 | 0.73 |
| **No Impairment** | 399 | 17 (338) | 14 to 19 |  |  |
| **Executive Functioning Domain** | | | | | |
| **Cognitive Flexibility Impairment** | 67 | 15 (232.5) | 9 to 20 | -0.1059 | 0.92 |
| **No Impairment** | 409 | 17 (338) | 14 to 19 |  |  |
|  |  |  |  |  |  |
| **Verbal Fluency Impairment** | 42 | 15.5 (118.5) | 9.5 to 25 | -0.1951 | 0.85 |
| **No Impairment** | 434 | 17 (338) | 14 to 19 |  |  |

P-values are based on Mann-Whitney tests

* P < 0.05

Supplemental Table 4c. Absolute Difference in Measures of Sleep Efficiency by Categorical Predictors

|  | **N** | **Median (range); %** | **95% CL** | **Z** | **P-value** |
| --- | --- | --- | --- | --- | --- |
| **Female** | 253 | 12.7 (67.6) | 10.8 to 15.0 | -2.4605 | 0.01* |
| **Male** | 221 | 9.1 (71.1) | 7.9 to 10.6 |  |  |
|  |  |  |  |  |  |
| **Non-Hispanic White** | 415 | 10.6 (71.1) | 9.2 to 12.1 | 0.2611 | 0.79 |
| **Other** | 59 | 11.0 (41.0) | 8.5 to 14.0 |  |  |
|  |  |  |  |  |  |
| **Psychological Health** | | | | | |
| **Anxiety** | 25 | 13.2 (69.1) | 9.8 to 19.2 | 1.4206 | 0.16 |
| **No Anxiety** | 411 | 10.4 (67.6) | 9.1 to 12.0 |  |  |
|  |  |  |  |  |  |
| **Depression** | 27 | 14.0 (70.1) | 10.8 to 22.6 | 2.1541 | 0.03* |
| **No Depression** | 409 | 10.1 (67.6) | 9.0 to 11.6 |  |  |
| **Sleep Quality** | | | | | |
| **Poor Sleep Quality** | 311 | 15.9 (71.0) | 13.9 to18.6 | -11.4379 | <0.0001* |
| **Good Sleep Quality** | 162 | 5.1 (26.8) | 4.4 to 6.2 |  |  |
|  |  |  |  |  |  |
| **Fatigue** | | | | | |
| **Severe Fatigue** | 61 | 14.0 (71.0) | 9.5 to 24.3 | 1.7797 | 0.08 |
| **< Severe Fatigue** | 382 | 10.1 (67.6) | 9.1 to 11.5 |  |  |
|  |  |  |  |  |  |
| **Neurocognitive Impairment** | | | | | |
| **Memory Domain** | | | | | |
| **Memory Impairment** | 40 | 14.0 (48.8) | 7.9 to 19.4 | 0.6930 | 0.49 |
| **No Impairment** | 434 | 10.5 (71.1) | 9.5 to 12.0 |  |  |
|  | | | | | |
| **Inattention Impairment** | 59 | 10.7 (43.7) | 8.0 to 19.2 | 0.4022 | 0.69 |
| **No Impairment** | 415 | 10.6 (71.1) | 9.5 to 12.1 |  |  |
|  |  |  |  |  |  |
| **Sustained Attention Impairment** | 77 | 12.8 (71.1) | 8.0 to 20 | 0.9482 | 0.34 |
| **No Impairment** | 397 | 10.5 (67.6) | 9.5 to 12.0 |  |  |
| **Executive Functioning Domain** | | | | | |
| **Cognitive Flexibility Impairment** | 66 | 11.3 (42.9) | 8.6 to 14.3 | -0.0155 | 0.99 |
| **No Impairment** | 408 | 10.6 (71.1) | 9.4 to 12.5 |  |  |
|  |  |  |  |  |  |
| **Verbal Fluency Impairment** | 42 | 8.2 (41.3) | 6.5 to 16.0 | -0.9782 | 0.33 |
| **No Impairment** | 432 | 10.9 (71.1) | 9.7 to 12.5 |  |  |

P-values are based on Mann-Whitney tests

* P < 0.05

Supplemental Table 5. General Linear Models Examining the Associations between Survivor Late Effects and Raw Difference Between Self-Reported and Actigraphic Sleep Measures Among Lower Self-Reporters (n=263-289)

|  | **Lower Self-Reporters: Self-Reported and Actigraphic Measure** | | | | | | | |
| --- | --- | --- | --- | --- | --- | --- | --- | --- |
|  | **Sleep Duration**  **(minutes)** | |  | **Sleep Onset Latency**  **(minutes)** | |  | **Sleep Efficiency**  **(%)** | |
| **Survivor Late Effects** | *Β*  *(95% CI)* | *P-value* |  | *Β*  *(95% CI)* | *P-value* |  | *Β*  *(95% CI)* | *P-value* |
| Anxiety^†^ | -0.36  *(-41.76 – 45.95)* | 0.99 |  | -2.96  *(-26.75 – 20.84)* | 0.81 |  | -1.28  *(-9.07 – 6.51)* | 0.75 |
| Depression^†^ | 2.79  *(-37.51* – *43.09)* | 0.89 |  | 33.75  *(10.87 – 56.63)* | 0.01* |  | 1.66  *(-5.35 – 8.68)* | 0.64 |
| Severe fatigue^‡^ | 21.99  *(-1.97 – 45.94)* | 0.07 |  | 2.68  *(-12.32 – 17.69)* | 0.72 |  | 1.36  *(-3.04 – 5.75)* | 0.54 |
| Poor sleep quality^§^ | 45.06  *(26.09 – 64.02)* | 0.0001* |  | 18.55  (7.23 *–* 29.87) | 0.01* |  | 13.15  *(9.74 – 16.57)* | 0.0001* |
| Memory impairment^¶^ | 31.39  *(1.71 – 61.06)* | 0.04* |  | -5.67  *(-26.63 – 15.29)* | 0.59 |  | -0.17  *(-5.50 – 5.15)* | 0.95 |
| Inattention impairment^¶^ | -1.75  (-26.97 – 23.47) | 0.89 |  | -1.51  *(-18.94 – 13.43)* | 0.86 |  | -0.09  *(-5.14 – 4.95)* | 0.97 |
| Sustained attention impairment^¶^ | 6.78  *(-16.51 – 30.08)* | 0.57 |  | 7.28  *(-8.29 – 22.86)* | 0.36 |  | 1.76  *(-2.75 – 6.27)* | 0.44 |
| Cognitive flexibility impairment^¶^ | -0.08  *(-26.28 – 26.12)* | 0.99 |  | -3.34  *(-20.08 – 13.40)* | 0.69 |  | 0.32  *(-4.14 – 4.78)* | 0.89 |
| Verbal fluency impairment^¶^ | 2.48  *(-29.30 – 34.26)* | 0.88 |  | 4.61  *(-14.71 – 23.94)* | 0.64 |  | -1.13  *(-6.65 – 4.38)* | 0.69 |
| **Demographic Covariates** |  |  |  |  |  |  |  |  |
| Age at evaluation (per one year) | 0.21  *(-0.71 – 1.13)* | 0.66 |  | 0.01  *(-0.54 – 0.56)* | 0.97 |  | -0.01  *(-0.17 – 0.15)* | 0.93 |
| Sex^††^ | 2.55  *(-13.71 – 18.81)* | 0.76 |  | 3.26  *(-6.43 – 12.94)* | 0.51 |  | 0.30  *(-2.64 – 3.23)* | 0.84 |
| Race/ethnicity^‡‡^ | -33.18  *(-56.90* – *-9.47)* | 0.01* |  | -2.59  *(-18.60 – 13.43)* | 0.75 |  | -2.66  *(-6.70 – 1.37)* | 0.20 |

^†^Anxiety and depression were defined as a T-score ≥63 on the Brief Symptom Inventory-18 measure for the depression or anxiety subscale, reference group = no anxiety or depression

^‡^Severe fatigue was defined as a score >30 on the FACIT Fatigue measure, reference group ≤ severe fatigue

^§^Poor sleep quality was defined as a score >5 on the Pittsburgh Sleep Quality Index, reference group = normal sleep quality

^¶^ Neurocognitive impairment defined as z-score ≤ 1.5 standard deviations below the mean, reference group= no impairment

^††^Females, reference group = males

^‡‡^“Other”, reference group = non-Hispanic, white

^*^ P < 0.05

Supplemental Table 6. General Linear Models Examining the Associations between Survivor Late Effects and Raw Difference Between Self-Reported and Actigraphic Sleep Measures Among Higher Self-Reporters (n=116-142)

|  | **Higher Self-Reporters: Difference between Self-Reported and Actigraphic Measure** | | | | | | | |
| --- | --- | --- | --- | --- | --- | --- | --- | --- |
|  | **Sleep Duration**  **(minutes)** | |  | **Sleep Onset Latency**  **(minutes)** | |  | **Sleep Efficiency**  **(%)** | |
| **Survivor Late Effects** | *Β*  *(95% CI)* | *P-value* |  | *Β*  *(95% CI)* | *P-value* |  | *Β*  *(95% CI)* | *P-value* |
| Anxiety^†^ | -27.43  *(-79.91 – 25.05)* | 0.30 |  | 19.75  *(-6.01 – 45.50)* | 0.13 |  | 2.80  *(-1.50 – 7.09)* | 0.20 |
| Depression^†^ | -13.66  *(-68.81 – 41.49)* | 0.62 |  | -2.67  *(-29.08 – 23.73)* | 0.84 |  | 1.21  *(-4.31 – 6.73)* | 0.66 |
| Severe fatigue^‡^ | 66.73  *(29.66 – 103.79)* | 0.001* |  | -0.64  *(-14.86 – 13.58)* | 0.93 |  | -2.88  *(-6.02 – 0.26)* | 0.07 |
| Poor sleep quality^§^ | -20.52  *(-43.71 – 2.67)* | 0.08 |  | 9.37  *(0.12 – 18.63)* | 0.05* |  | 2.33  *(0.36 – 4.31)* | 0.02* |
| Memory impairment^¶^ | 46.90  *(4.20 – 89.59)* | 0.03* |  | -1.66  *(-15.95 – 12.62)* | 0.82 |  | -1.90  *(-5.88 – 2.08)* | 0.35 |
| Inattention impairment^¶^ | -4.33  *(-44.89 – 36.23)* | 0.83 |  | 6.75  *(-6.74 – 20.23)* | 0.32 |  | 0.55  *(-2.59 – 3.69)* | 0.73 |
| Sustained attention impairment^¶^ | -3.80  *(-37.78 – 30.18)* | 0.83 |  | -7.44  *(-19.73 – 4.86)* | 0.23 |  | 2.21  *(-0.47 – 4.89)* | 0.11 |
| Cognitive flexibility impairment^¶^ | 6.67  *(-25.98 – 39.32)* | 0.69 |  | 8.13  *(-4.15 – 20.41)* | 0.19 |  | -0.46  *(-3.64 – 2.71)* | 0.77 |
| Verbal fluency impairment^¶^ | -24.41  *(-58.62 – 9.80)* | 0.16 |  | -0.94  *(-15.40 – 13.52)* | 0.90 |  | 3.20  *(-0.12 – 6.52)* | 0.06 |
| **Demographic Covariates** |  |  |  |  |  |  |  |  |
| Age at evaluation (per one year) | -1.29  *(-2.55 – -0.02)* | 0.05* |  | -0.03  *(-0.58 – 0.51)* | 0.91 |  | 0.02  *(-0.10 – 0.14)* | 0.75 |
| Sex^††^ | -26.07  *(-48.05 – -4.09)* | 0.02* |  | -1.54  *(-11.31 – 8.23)* | 0.76 |  | -0.97  *(-2.93 – 0.99)* | 0.33 |
| Race/ethnicity^‡‡^ | 18.47  *(-12.38 – 49.32)* | 0.24 |  | 13.26  *(1.50 – 25.03)* | 0.03* |  | 2.23  *(-1.16 – 5.63)* | 0.19 |

^†^Anxiety and depression were defined as a T-score ≥63 on the Brief Symptom Inventory-18 measure for the depression or anxiety subscale, reference group = no anxiety or depression

^‡^Severe fatigue was defined as a score >30 on the FACIT Fatigue measure, reference group ≤ severe fatigue

^§^Poor sleep quality was defined as a score >5 on the Pittsburgh Sleep Quality Index, reference group = normal sleep quality

^¶^ Neurocognitive impairment defined as z-score ≤ 1.5 standard deviations below the mean, reference group= no impairment

^††^Females, reference group = males

^‡‡^“Other”, reference group = non-Hispanic, white

^*^ P < 0.05
